# Factors influencing diagnosis delay of advanced breast cancer in Moroccan women

**DOI:** 10.1186/s12885-016-2394-y

**Published:** 2016-06-07

**Authors:** A. Maghous, F. Rais, S. Ahid, N. Benhmidou, K. Bellahamou, H. Loughlimi, E. Marnouche, S. Elmajjaoui, H. Elkacemi, T. Kebdani, N. Benjaafar

**Affiliations:** Department of radiotherapy, National Institute of Oncology, Mohammed V University in Rabat, Rabat, Morocco; Department of Medical Oncology, National Institute of Oncology, Mohammed V University in Rabat, Rabat, Morocco; Laboratory of epidemiology and clinical research, School of medicine and pharmacy of Rabat, Mohammed V University in Rabat, Rabat, Morocco

**Keywords:** Breast cancer, Diagnosis delay, Patient delay, System delay

## Abstract

**Background:**

Delay in the diagnosis of breast cancer in symptomatic women of 3 months or more is associated with advanced stage and low survival. We conducted this study to learn more about the extent and reasons behind diagnosis delay of advanced breast cancer in Moroccan women.

**Methods:**

A group of patients with advanced breast cancer were interviewed at the National Institute of Oncology in Rabat during the period from February to December 2014. Diagnosis delay was devised into patient delay and system delay. Patient delay was defined as time from first symptoms until first medical consultation. System delay was defined as time from first presentation to a health care provider until definite diagnosis or treatment. Prospective information and clinical data were collected on a form during an interview with each patient and from medical records.

**Results:**

In all, 137 patients were interviewed. The mean age of women was 48.3 ± 10.4 years. The median of consultation time was 6[4,12] months and the median of diagnosis time was 1[1,3] months. Diagnosis delay was associated to a personal reason in 96 (70.1 %) patients and to a medical reason in 19 (13.9 %) patients. A number of factors predicted diagnosis delay: symptoms were not considered serious in 66 (55.9 %) patients; traditional therapy was applied in 15 (12.7 %) patients and fear of cancer diagnosis and/or treatment in 14 (11.9 %) patients. A use of traditional methods was significantly associated with rural residence and far away from basic health center (*p* = 0.000). Paradoxically, a family history of breast cancer was significantly higher in who report a fear of cancer diagnosis and/or treatment to diagnosis delay (*p* < 0.001). Also, a significantly higher risk of more than 6 months delay was found among rural women (*P* = 0.035) and women who live far away from specialized care center (*P* = 0.001).

**Conclusions:**

Diagnosis delay is very serious problem in Morocco. Diagnosis delay was associated with complex interactions between several factors and with advanced stages. There is a need for improving breast cancer information in our populations and training of general practitioners to reduce advanced breast cancer by promoting early detection.

**Electronic supplementary material:**

The online version of this article (doi:10.1186/s12885-016-2394-y) contains supplementary material, which is available to authorized users.

## Background

Breast cancer is the most frequently occurring cancer in women all over the world with nearly a half million deaths each year [1]. It is now the most common cancer both in developed and developing regions. In Morocco, breast cancer represents a serious public health problem. It’s the first cancer among women with a standardized incidence of 49.2 for an average age of 50 years according to 2006–2008 data reported by the Rabat Cancer Registry [2].

Longer waiting times prior to diagnosis and the initiation of therapy are likely to result in advanced disease and low survival [3–5]. The delayed diagnosis is more responsible rather than the disease itself in causing mortality of the patient, as early diagnosis and treatment is associated with better prognosis when compared to worse outcomes related to significantly delayed diagnosis. For example, studies in developed and developing countries reported that a diagnosis delay of more than 3 months occurred in 14–53 % of cases [6–15]. Low socio-economic status, minority ethnicity and young age were associated with a longer duration of symptoms [8]. Morocco shares the same panorama of other developing countries, with the majority of breast cancer patients starting treatment in advanced stages of the disease [6]. Until 2005, date of creation of Lalla Salma Foundation against cancer (ALSC), it was commonly assumed that this is due to the populations lack of information and to deficient coverage of screening programs. Nevertheless, the reasons behind always diagnostic delay of advanced breast cancer in Morocco are varied.

Traditionally, breast cancer diagnosis delay has been classified in two types: (1) patient delay, the interval between the discovery of symptoms and the first medical consultation; and (2) system delay, the interval between first consultation and definite diagnosis or treatment. Prolonged delays usually defined as intervals greater than 12 weeks [7]. We conducted this study to learn more about the extent and reasons behind diagnosis delay of advanced breast cancer in Moroccan women.

## Methods

### Population and study sites

This cross-sectional study was conducted during the period from February 2014 to December 2014 at the National Institute of Oncology in Rabat. During that time a group of patients with advanced breast cancer (T3 or T4 or metastatic at the time of diagnosis) were interviewed at the institute after obtaining oral consent from each patient. Participants were recruited either after a computed Tomography (CT) simulation of radiotherapy or during follow-up in the outpatient department. Women were excluded if their breast abnormality was discovered through breast screening or if the delay was less than three months.

### Data collection

Structured face-to-face questionnaire (Additional file 1) were conducted by previously trained resident interviewers who were not involved in the clinical management of the patients. The supplemented information has been filled from patient medical records. Data was collected using a well structured checklist containing important study parameters witch derived from the existing literature on delayed presentation in breast cancer.

The record collection includes social and demographic data: age at presentation (years), area of residence (rural or urban), marital status (single, married, widowed or divorced), occupation (no occupation, house wife, retired, full time or part time employed), patient (and spouse) educational level (illiterate, primary, secondary or higher education), number of dependent children (none, 1–3 or ≥ 4) and number of dependents (none, 1–2 or ≥ 3), distance from basic health center (<5 km or ≥5 km), distance from specialized care (<100 km or ≥100 km).

Data includes also first clinical presenting symptoms (breast or axillary lump, skin changes, breast pain, nipple discharge, bone pain…), breast self-examination, family history of breast cancer, tumor size (cm) and classification of disease (TNM). The date of first symptoms, date of first consultation and date of first diagnosis were collected to calculate consultation time (the time taken to visit the general practitioner after the first symptoms) and diagnosis time (the time measured from the date of the first medical consultation to the date of final breast cancer diagnosis based on pathological examination).

### Patient and system delay

Patient delay refers to delays occurring between the discovery of symptoms and the first medical consultation, and the most accepted threshold to establish it is three months. System delay is that which takes place between the first medical consultation and the definite diagnosis or treatment, and the most accepted threshold is one month.

Factors related to patient delay (not attributed the symptoms to cancer, fear of cancer diagnosis and/or treatment, financial constraints, competing life priorities, embarrassment about having a breast examination, use of traditional methods…) and/or system delay (patient reassured after negative physical breast examination, mammography misinterpreted, non-specific medical treatment without control, a negative fine-needle aspiration biopsy, surgical excision without pathological examination, not oriented to specialized service, lack of information, appointment delay …) were collected.

### Statistical analysis

Statistical analysis of the data was carried out by the SPSS for Windows 13.0 (SPSS, Inc., Chicago, IL, USA). Qualitative variables were presented as number and percentages. Quantitative variables were presented as mean ± standard deviation for variables with normal distribution, and as median and interquartile range (IQR) for variables with skewed distributions. Chi2 tests and Fisher test were used to identify factors associated with different delays. A multivariate logistic regression was used to determine factors associate with longer delay. In all tests, the values *p* < 0.05 were regarded statistically significant.

## Results

### Study population

In all, 137 patients were interviewed. The mean age of women was 48.3 ± 10.4 years. Eighty Nine patients (65 %) resided in urban area. Seventy seven patients (56.2 %) were married. One hundred and fifteen patients (83.9 %) were illiterate. Fifty two (38 %) did not have any dependent children. Only twenty nine patients (20.4 %) had a family history of breast cancer. Breast or axillary lump was a first clinical presentation in 90 (65.7 %) of patients. Tumor size was greater than 5 cm in 91 (77.8 %) of patients. The median of consultation time was 6[4, 12] months and the median of diagnosis time was 1[1, 3] months. Other characteristics of the study population (*n* = 137) are shown in Tables 1 and 2.

**Table 1:** Social demographic characteristics of the patients (*n* = 137)

		N (%)
Age at presentation (years)	Mean (±SD)	48.3 ± 10.4
Area of residence	Rural	48 (35)
	Urban	89 (65)
Marital status	Single	26 (19)
	Married	77 (56.2)
	Widowed or Divorced	34 (24.8)
Occupation	No occupation	23 (16.8)
	House wife	102 (74.5)
	Full time employed	10 (7.3)
	Part time employed	2 (1.5)
Patient educational level	Illiterate	115 (83.9)
	Primary	14 (10.2)
	Secondary/Higher	8 (5.8)
Spouse educational level (*n* = 77)	Illiterate	49 (63.6)
	Primary	20 (26)
	Secondary/Higher	8 (10.4)
Number of dependent children	None	52 (38)
	1–3	51 (37.2)
	≥4	34 (24.8)
Number of dependents	None	35 (25.5)
	1–2	86 (62.8)
	≥3	16 (11.7)
Distance from basic health center (km)	<5	101 (73.7)
	≥5	36 (26.3)
Distance from specialized care (km)	<100	76 (55.5)
	≥100	61 (44.5)

**Table 2:** Medical history of the patients (*n* = 137)

		N (%)
First clinical presentations	Lump	90 (65.7)
	Skin changes	21 (15.3)
	Breast pain	16 (11.7)
	Nipple discharge	6 (4.4)
	Bone pain	4 (2.9)
Breast self-examination	No	115 (83.9)
	Yes	22 (16.1)
Family history of breast cancer	No	109 (79.6)
	Yes	28 (20.4)
Consultation time or patient delay (months)	Median [IQR]	6[4, 12]
Diagnosis time or system delay (months)	Median [IQR]	1[1, 3]
Total diagnosis delay (months)	Median [IQR]	10[5.7, 13]
Tumor characteristics		
Tumor size (*n* = 117)	≤2 cm	3 (2.6)
	3–5 cm	23 (19.7)
	>5 cm	91 (77.8)
TNM classification		
T	T2	5 (3.6)
	T3	71 (51.8)
	T4	61 (44.5)
N	N0	48 (35)
	N1	51 (37.2)
	N2	18 (13.1)
	N3	20 (14.6)
M	M0	114 (83.2)
	M1	23 (16.8)

*Abbreviations: IQR* interquartile range

### Diagnosis delay and associated factors

Table 3 reports the causes of diagnosis delay and associated factors. Among the 137 patients interviewed in this study, 96 (70.1 %) reported a personal reason to diagnosis delay. From 66 (55.9 %) of patients they were not attributed the first symptoms presentations to breast cancer, 49 (41.5 %) of them thought that the absence of pain cancer diagnosis remains unlikely and 12 (10.2 %) of them were breastfeeding so they put all of breast symptoms on the account of its complications. Traditional therapy was applied in 15 (12.7 %) of the patients. On the other hand, 19 (13.9 %) of patients interviewed reports a medical reason to diagnosis delay. According to patient perception 10 (24.4 %) were inappropriately reassured after negative physical breast examination. Other factors are reported in Table 3. Often the factors related to diagnosis delay were intricate. Twenty two (16.1 %) patients report at the same time a personal and medical reason to diagnosis delay.

**Table 3:** Diagnosis delay and associated factors

	N (%)
Causes of Diagnosis Delay (*n* = 137)	
Cause related to patient	96 (70.1)
Cause related to health system	19 (13.9)
Cause related to patient and system	22 (16.1)
Factors related to patient delay (*n* = 118)	
Symptoms not attributed to cancer	66 (55.9)
Lack of information	49 (41.5)
Symptoms related to breastfeeding	12 (10.2)
Symptoms related to benign breast disease	5 (4.2)
Use of traditional methods	15 (12.7)
Fear of cancer diagnosis and/or treatment	14 (11.9)
Financial constraints	8 (6.8)
Competing life priorities	8 (6.8)
Embarrassment about having a breast examination	7 (5.9)
Factors related to system delay (*n* = 41)	
Negative physical breast examination	10 (24.4)
Non-specific medical treatment without control	8 (19.5)
A negative fine-needle aspiration biopsy	8 (19.5)
Appointment delay	8 (19.5)
Mammography misinterpreted	4 (9.8)
Surgical excision without pathological examination	1 (2.4)
Lack of information	2 (4.9)

To better characterize these different factors we conducted a comparative study of the social demographic characteristics and medical history of the patients according to the principal factors related to diagnosis delay and according to diagnosis time, Tables 4 and 5 reports this results respectively.

**Table 4:** Comparison of patients according to the principal cause of delay

		Symptoms not attributed to cancer (*n* = 53)	Traditional methods (*n* = 13)	Fear of cancer (*n* = 11)	System delay (*n* = 19)	*P* value
Social demographic characteristics of the patients						
Age at presentation (years)	Mean (±SD)	47.74 ± 9.77	45.46 ± 9.56	44 ± 11.19	47.47 ± 10.4	0.852
Area of residence	Rural	18 (34)	**12 (92.3)**	1 (9.1)	4 (21.1)	**0.001**
	Urban	**35 (66)**	1 (7.7)	**10 (90.9)**	**15 (78.9)**	
Marital status	Single	10 (18.9)	3 (23.1)	5 (45.4)	5 (26.3)	0.535
	Married	30 (56.6)	9 (69.2)	5 (45.4)	10 (52.6)	
	Widowed/Divorced	13 (24.5)	1 (7.7)	1 (9.1)	4 (21.1)	
Occupation	No occupation	9 (17)	3 (23.1)	4 (36.4)	4 (21.1)	0.234
	House wife	41 (77.4)	10 (76.9)	5 (45.5)	12 (63.2)	
	Employed	3 (5.7)	0 (0.0)	2 (18.2)	3 (15.8)	
Patient educational level	Illiterate	45 (84.9)	13 (100)	8 (72.7)	15 (78.9)	0.222
	Literate	8 (15.1)	0 (0.0)	3 (27.3)	4 (21.1)	
Spouse educational level	Illiterate	19 (63.3)	9 (100)	3 (60)	5 (50)	0.077
	Literate	11 (36.7)	0 (0.0)	2 (40)	5 (50)	
Number of dependent children	None	20 (37.7)	5 (38.5)	7 (63.6)	8 (42.1)	0.813
	1–3	21 (39.6)	5 (38.5)	2 (18.2)	8 (42.1)	
	≥4	12 (22.6)	3 (23.1)	2 (18.2)	3 (15.8)	
Number of dependents	None	13 (24.5)	3 (23.1)	4 (36.4)	5 (26.3)	0.895
	1–2	35 (66)	9 (69.2)	5 (45.5)	12 (63.2)	
	≥3	5 (9.4)	1 (7.7)	2 (18.2)	2 (10.5)	
Distance from basic health center (km)	<5	**42 (79.2)**	3 (23.1)	**9 (81.8)**	**15 (78.9)**	**0.001**
	≥5	11 (20.8)	**10 (76.9)**	2 (18.2)	4 (21.1)	
Distance from specialized care (km)	<100	**33 (62.3)**	0 (0.0)	**8 (72.7)**	**14 (73.7)**	**<0.001**
	≥100	20 (37.7)	**13 (100)**	3 (27.3)	5 (26.3)	
Medical history of the patients						
First clinical presentations	Lump	31 (58.5)	10 (76.9)	7 (63.6)	13 (68.4)	0.655
	Other	22 (41.5)	3 (23.1)	4 (36.4)	6 (31.6)	
Breast self-examination	No	**50 (94.3)**	**13 (100)**	**9 (81.8)**	7 (36.8)	**<0.001**
	Yes	3 (5.7)	0 (0.0)	2 (18.2)	**12 (63.2)**	
Family history of breast cancer	No	**50 (94.3)**	**12 (92.3)**	2 (18.2)	**11 (57.9)**	**<0.001**
	Yes	3 (5.7)	1 (7.7)	**9 (81.8)**	8 (42.1)	

Numbers in bodface = significant results

**Table 5:** Comparison of patients according to diagnosis delay

		Diagnosis delay		Bivariated analysis			Multivariated analysis		
		≤6 months (*n* = 39)	>6 months (*n* = 98)	OR	95 % CI	*P* value	OR	95 % CI	*P* value
Social demographic characteristics of the patients									
Age at presentation (years)	Mean (±SD)	47.7 ± 10.39	48.53 ± 10.47	1.00	0.972–1.04	0.699			
Area of residence	Rural	2 (5.1)	46 (46.9)	**16.36**	**3.74–71.69**	**<0.001**	**9.73**	**1.17–80.93**	**0.035**
	Urban	37 (94.9)	52 (53.1)	1(Ref)					
Marital status	Single	9 (23.1)	17 (17.3)	1(Ref)					
	Married	17 (43.6)	60 (61.2)	1.87	0.71–4.93	0.207			
	Widowed/Divorced	13 (33.3)	21 (21.4)	0.85	0.29–2.48	0.773			
Occupation	No occupation	8 (20.5)	15 (15.3)	1(Ref)					
	House wife	26 (66.7)	76 (77.6)	1.56	0.59–4.10	0.368			
	Employed	5 (12.8)	7 (7.1)	0.75	0.18–3.13	0.689			
Patient educational level	Illiterate	17 (43.6)	98 (100)	9 10^9^	0–< 0.001	0.999			
	Primary	14 (35.9)	0 (0.0)	1	0–< 0.001	1.000			
	Secondary/Higher	8 (20.5)	0 (0.0)	1(Ref)					
Spouse educational level (*n* = 77)	Illiterate	5 (29.4)	44 (73.3)	5.28	0.96–29.02	0.056			
	Primary	9 (52.9)	11 (18.3)	0.73	0.14–3.94	0.718			
	Secondary/Higher	3 (17.6)	5 (8.3)	1(Ref)					
Number of dependent children	None	17 (43.6)	35 (35.7)	1(Ref)					
	1–3	13 (33.3)	38 (38.8)	1.42	0.60–3.34	0.422			
	≥4	9 (23.1)	25 (25.5)	1.35	0.52–3.51	0.540			
Number of dependents	None	11 (28.2)	24 (24.5)	1(Ref)					
	1–2	22 (56.4)	64 (65.3)	1.33	0.56–3.16	0.513			
	≥3	6 (15.4)	10 (10.2)	0.76	0.22–2,64	0.670			
Distance from basic health center (km)	<5	34 (87.2)	67 (68.4)	1(Ref)					
	≥5	5 (12.8)	31 (31.6)	**3.15**	**1.12–8.82**	**0.029**	**0.09**	**0.01–0.90**	**0.040**
Distance from specialized care (km)	<100	37 (94.9)	39 (39.8)	1(Ref)					
	≥100	2 (5.1)	59 (60.2)	**27.99**	**6.38–122.85**	**<0.001**	**32.77**	**4.42–242.92**	**0.001**
Medical history of the patients									
First clinical presentations	Lump	27 (69.2)	63 (64.3)	1(Ref)					
	Other	12 (30.8)	35 (35.7)	1.25	0.56–2.77	0.583			
Breast self-examination	No	27 (69.2)	88 (89.8)	**3.91**	**1.52–10.05**	**0.005**	1.20	0.37–3.89	0.765
	Yes	12 (30.8)	10 (10.2)	1(Ref)					
Family history of breast cancer	No	23 (59)	86 (87.8)	**4.99**	**2.07–12.00**	**<0.001**	**4.46**	**1.39–14.32**	**0.012**
	Yes	16 (41)	12 (12.2)	1(Ref)					

*Abbreviations: OR* odds ratio, *95 CI = 95 %* confidence interval

Numbers in bodface = significant results

According to the principal factors related to diagnosis delay (Table 4), a use of traditional methods was significantly associated with rural residence and far away from basic health and specialized care center (*p* = 0.001). Breast self-examination was significantly practical in patients who report a medical reason to diagnosis delay (*p* < 0.001). Paradoxically, a family history of breast cancer was significantly higher in who report a fear of cancer diagnosis and/or treatment to diagnosis delay (*p* < 0.001).

According to total diagnosis delay; Table 5 shows the result of univariate and multivariate logistic regression analysis. There was a significant risk for longer delay more than six months among rural women (univariate OR, 16.36, 95 % CI,3.74–71.69; *P* < 0.001; multivariate OR, 9.73; 95 % CI, 1.17–80.93; *P* = 0.035), women who live far away from specialized care center (univariate OR, 27.99, 95 % CI, 6.38-122.85; *P* < 0.001; multivariate OR, 32.77; 95 % CI, 4.42–242.92; *P* = 0.001) and women without family history of breast cancer (univariate OR, 4.99, 95 % CI, 2.07–12.00; *P* < 0.001; multivariate OR, 4.46; 95 % CI, 1.39–14.32; *P* = 0.012). Although univariate analysis suggested a significant risk among women who did not report breast self examination (OR, 3.91, 95 % CI, 1.52–10.05; *P* = 0.005), but this was not significant in the multivariate model (OR, 1.20; 95 % CI, 0.37–3.89; *P* = 0.765). No significant differences were found among the other variables studied.

## Discussion

In Morocco, breast cancer is the most prevalent cancer in women and a major public health problem. Also, they it causes a significant add up of deaths due to the delay in their its diagnosis according to the Rabat Cancer Registry. This is the first study conducted in our country to learn more about the extent and reasons behind diagnosis delay of advanced breast cancer. Through understanding the causes of delay it may be possible to reduce delays and to improve early diagnosis.

Patient’s delay has a very important contribution to a delayed diagnosis (70.1 %). The median duration of delay in our study was higher than in developed [8–10] or developing countries [11–15]. About 71.5 % of symptomatic patients had a delay of more than six months. Perhaps this trend can be attributed to advanced stage of the study population witch correlate with more diagnosis delay [16–19]. Living in rural area or in far distance from specialized care was a significant predictor of longer delay. Other studies has been shown that women who live in larger households may have to care for children or other dependents and thus are at higher risk to present with late stage breast cancer [20, 21]. Also, being less educated (83.9 %) or absence of regular breast self examination (83.9 %) within our patients suggested a possible predictor of longer delay. The role of education and knowledge in decreasing delay has been confirmed in other studies [22–24]. A multinational analysis shows that women who made breast self examination were more educated and tended to seek medical care more rapidly [25].

In the present study the nature of the first symptom had no association with patient delay. Breast lump was a first alarm symptom in majority of our patients (65.7 %). However, women did not have sufficient background knowledge regarding this symptom and therefore the discovery of a breast lump did not reduced the patient delay witch contrasted with other studies [18, 24, 25]. The findings suggest that women need to be educated about the different types of breast cancer symptoms, especially the most frequent symptom as well as encouragement to seek medical advice if a symptom is ambiguous.

The most frequent reasons given by women for a delayed consultation were their perception regarding the symptoms to be harmless and temporary, adding, that the absence of pain cancer diagnosis remains unlikely. This demonstrates poor knowledge of our females regarding importance of these warning signs and symptoms of breast cancer, that how much severity these symptoms can attain with the passage of time, being irreversible and even proving to be fatal in late stages. This result was consistent with a similar study conducted in Tunisia [11], in Libya [12], in Nigeria [13] and others [7, 26].

Despite of the fact that awareness in our population regarding medical health care has increased as compared to past especially after creation of Lalla Salma Foundation against cancer (ALSC), still a large part of women initially prefer use of traditional methods, which represent 12.7 % of the respondents in current study, this is consistent with a regional study that found antecedent use of unconventional and alternative therapies before seeking any medical advice as an important reason for Patient’s delay [11, 12]. Most patients took alternative treatment as means to avoid surgery. Some patients believed that there were no effective treatments for breast cancer, or that traditional medicines are more effective than modern drugs. This conviction was significantly associated with rural residence and far away from basic health and specialized care center. Low socioeconomic status was one of the factors for delay in diagnosis [27].

This study showed that a fear of cancer diagnosis and/or treatment (11.9 %) paradoxically caused delays as in developing countries [11–13]; especially in who report a family history of breast cancer (81.8 %). Negative information, such as side effects and expected toxicity of chemotherapy led to fear and refusal of therapy. Fear of divorce or remarriage of the husband could lead some women to decide not to get their symptoms diagnosed if they suspected breast cancer. Some patients also convinced that breast cancer could not be cured [28], so there was no point of having it diagnosed and treated. Diagnosis delay was also related to a belief that mastectomy causes disfigurement and disability [29].

Interestingly, the present study revealed that even breast lump was a first clinical presentation; it was not always well assessed within nongynecologists doctors. Our findings indicate that 24.4 % of respondents were inappropriately reassured after the first visit to the general practitioners that a lump can be considered benign without biopsy. This is a false attitude. In this study, this attitude was an important reason to the system delay. Similar results were reported by Goodson et al. [30]. The findings suggest that doctors also need to be more educated about the different types of breast cancer symptoms and their management.

The study did not demonstrate any significant association between age, marital status, occupation, number of dependents and delay in diagnosis. However, several studies have shown that older age is a predictor for patient delay but it remains controversial with marital status [31, 32]. Occupation and number of dependents and children had also a significant statistical correlation with patient delay [20, 21].

In limitations, that it is worth noting there were inherent in this study. The factors related to diagnosis delay were intricate. Additionally, most of the patients were from low socioeconomic status with majority of them being illiterate and so the results cannot be generalized to whole of the population.

## Conclusions

In conclusion, diagnosis delay is very important health problem in Morocco women with breast cancer which is associated with complex interactions between several factors. The study results do provide some understanding on the topic and found that almost all of those factors demonstrate a deficiency of sufficient knowledge, information and awareness in our population regarding this fatal disease. Moroccan women need more education on breast cancer especially who at higher risk of diagnosis delay, imparting adequate knowledge to its presenting signs and symptoms, also the necessity of regular self breast examination. Specific attention should be conducted to increase the awareness among general practitioners for improving breast cancer prognosis by early diagnosis and treatment.

## Abbreviations

ALSC, Lalla Salma Foundation against cancer; CT, computed Tomography; IQR, interquartile range

## Additional file

Additional file 1: Questionnaire. (DOC 32 kb)
