# Supplementary material for: Factors influencing diagnosis delay of advanced breast cancer in Moroccan women
Source: BMC Cancer. 2016 Jun 7;16:356. doi: 10.1186/s12885-016-2394-y (PMC4897875; doi:10.1186/s12885-016-2394-y)
Supplement: Additional file 1: — Questionnaire. (DOC 32 kb) [file 12885_2016_2394_MOESM1_ESM.doc]

**Questionnaire**

First and Last Name: ………………………………………………………

TNM : T……… N ……… M………. Tumor size :……………………..cm

Consultation time or patient delay (months): ………………………

Diagnosis time or system delay (months): ……………………………………………….…...

Social demographic characteristics of the patients

- Age: ……………………………..
- Area of residence : rural ❒ - urban❒
- Marital status : Single ❒ - Married ❒ - Widowed ❒ - Divorced ❒
- Occupation: No occupation ❒ - House wife ❒ - full time employed ❒ - part time employed ❒
- Patient educational level : Illiterate ❒ - Primary❒ - Secondary❒ - Higher ❒
- Spouse educational level : Illiterate ❒ - Primary❒ - Secondary❒ - Higher ❒
- Number of dependent children: ……………………………………………............................................
- Number of dependent: ……………………………………………………………………….
- Distance from basic health center (km): ………………………………………………………………
- Distance from specialized care (km): ………………………………………………………………..

First clinical presentations:

- Lump ❒ - Skin changes ❒ - Breast pain ❒ - Nipple discharge ❒
- Bone pain ❒
- Others :………………………………………………………………………………………………..

Family history of breast cancer: yes ❒ - no ❒

Factors related to patient delay

- No breast self-examination ❒
- Symptoms not attributed to cancer ❒ :

Lack of information ❒ - Symptoms related to breastfeeding ❒

Symptoms related to benign breast disease ❒

Others: ………………………………………………………

- Fear of cancer diagnosis and/or treatment ❒
- Financial constraints ❒
- Competing life priorities ❒…………………………………………………………………………….
- Use of traditional methods ❒
- Embarrassment about having a breast examination ❒
- Others: …………………………………….…………………...

Factors related to system delay

- Negative physical breast examination ❒
- Non-specific medical treatment without control ❒
- A negative fine-needle aspiration biopsy ❒
- Appointment delay ❒
- Mammography misinterpreted ❒
- Surgical excision without pathological examination ❒
- Lack of information ❒
- Others :……………………………………………………………………………….......................
